# A Nomogram for Predicting Surgical Timing in Neonates with Necrotizing Enterocolitis

**DOI:** 10.3390/jcm12093062

**Published:** 2023-04-23

**Authors:** Bo Shi, Leiting Shen, Wenchang Huang, Linghao Cai, Sisi Yang, Yuanyuan Zhang, Jinfa Tou, Dengming Lai

**Affiliations:** 1 Department of Neonatal Surgery, Children’s Hospital, Zhejiang University School of Medicine, National Clinical Research Center for Child Health, Hangzhou 310052, China; 2 Department of Pulmonology, Children’s Hospital, Zhejiang University School of Medicine, National Clinical Research Center for Child Health, Hangzhou 310052, China; 3 Binjiang Institute of Zhejiang University, Hangzhou 310053, China

**Keywords:** necrotizing enterocolitis, surgery, risk factor, nomogram, prediction model

## Abstract

Objective: To explore the surgical risk variables in patients with necrotizing enterocolitis (NEC) and develop a nomogram model for predicting the surgical intervention timing of NEC. Methods: Infants diagnosed with NEC were enrolled in our study. We gathered information from clinical data, laboratory examinations, and radiological manifestations. Using LASSO (least absolute shrinkage and selection operator) regression analysis and multivariate logistic regression analysis, a clinical prediction model based on the logistic nomogram was developed. The performance of the nomogram model was evaluated using the receiver operating characteristic (ROC) curve, calibration curves, and decision curve analysis (DCA). Results: A surgical intervention risk nomogram based on hypothermia, absent bowel sounds, WBC > 20 × 10^9^/L or < 5 × 10^9^/L, CRP > 50 mg/L, pneumatosis intestinalis, and ascites was practical, had a moderate predictive value (AUC > 0.8), improved calibration, and enhanced clinical benefit. Conclusions: This simple and reliable clinical prediction nomogram model can help physicians evaluate children with NEC in a fast and effective manner, enabling the early identification and diagnosis of children at risk for surgery. It offers clinical revolutionary value for the development of medical or surgical treatment plans for children with NEC.

## 1. Introduction

In neonates, necrotizing enterocolitis (NEC) is a frequent gastrointestinal emergency, especially in premature infants [1]. NEC has a complicated and variable etiology, and its pathogenic mechanism remains unknown. NEC is most commonly associated with preterm delivery. Lower birth weights are associated with an increased risk of NEC due to factors such as an immature gastrointestinal function, incomplete intestinal barrier, impaired gastrointestinal motility, impaired digestion and absorption function, and weakened intestinal immune function [2]. Currently, it is believed that the combined effects of genetic susceptibility, environment, intestinal susceptibility, and other factors on the intestinal tract of premature infants can result in intestinal mucosal injury, bacterial heterotopia, microthrombosis, and ultimately intestinal ischemia and necrosis [3]. Current research on the NEC mechanism focuses mostly on intestinal barrier function, overactive immunological response, intestinal flora imbalance, intestinal microcirculation abnormalities, and so on [4,5,6,7]. Children often show the first signs of feeding resistance through clinical indications, such as stomach retention, abdominal distension, and vomiting. Furthermore, children may experience increased bloating, bloody stools, apnea, abdominal flushing, and even intestinal perforation, which can lead to disseminated intravascular coagulation, respiratory failure, sepsis, infectious illness shock, and even death [1,8,9]. Children with NEC are characterized by an acute onset, rapid progression, and poor prognosis. Additionally, NEC is the leading cause of death in neonates in a neonatal intensive care unit (NICU), with an incidence of 5–10% and mortality of 20–30% in newborns with a birth weight less than 1500 g [10,11,12]. Therefore, striving for early detection, early diagnosis, and early treatment is crucial to improve the prognosis of NEC [13].

The clinical symptoms of children with NEC vary considerably between individuals. Therefore, children with suspected or confirmed NEC should fast and undergo gastrointestinal decompression as soon as feasible to minimize the pressure of intestinal material on the gut [14,15]. Antibiotics with a broad spectrum should be used empirically to combat infections, and parenteral feeding should be improved. About 30% to 50% of infants with necrotizing enterocolitis (NEC) require surgical intervention, mostly exploratory laparotomy and abdominal drainage [16,17,18]. However, precise clinical indicators and supplementary exams for the early detection of intestinal necrosis are still lacking, with the exception of pneumoperitoneum or intestinal perforation, which are acknowledged as unequivocal surgical indications for NEC [19]. For NEC patients without intestinal perforation, it is still difficult to determine the appropriate timing of surgery when the situation continues to deteriorate despite intensive nonsurgical treatment (intestinal necrosis has occurred but intestinal contents have not yet reached the abdominal cavity). As a result, the timing of surgery is more susceptible to departure due to the doctor’s reliance on his or her own clinical experience when establishing a diagnosis and prescribing treatment. In addition, intestinal necrosis and perforation caused by NEC typically worsen gradually. In this condition, the diseased intestine adheres to create a mass, and the gas in the intestinal cavity may not enter the abdominal cavity; therefore, the pneumoperitoneum sign is not a sensitive indicator of intestinal necrosis and perforation.

The diagnostic criteria and treatment of NEC are continuously updated and improved in accordance with the growing understanding of NEC disease and enhancements in imaging examination and laboratory detection technologies in medical institutions and among medical personnel of all levels. In addition, some studies have been published that investigate the optimal window for surgical intervention. Using a physical exam score for NEC (PE-NEC) to assess abdominal symptoms can assist in predicting whether children with NEC require surgical therapy [20]. Abdominal ultrasounds provide high diagnostic sensitivity and specificity for early intestine dynamic alterations and can also be used to monitor the superior mesenteric artery’s blood flow and assess the development of NEC. However, the therapeutic application and promotion of abdominal ultrasonography are dependent on the diagnostic expertise of ultrasound physicians [21,22]. Without professional training, clinicians are incapable of producing accurate diagnostic findings [23]. Based on an abdominal X-ray examination, Coursey et al. developed the Duke abdominal assessment scale (DAAS) to determine whether surgical exploration is necessary [24]. Tepas et al. based the time of operation on the frequency of seven clinical measurements of metabolic derangement (MD7) [25,26]. However, it is more crucial that we determine the severity of NEC and the time of operation based on systemic symptoms, abdominal signs, and supplementary examination indicators in the advanced disease stage. Therefore, we conducted a single-center analysis of the clinical data of hospitalized children with NEC to identify and validate the characteristics of considerable risk variables that can be used to determine whether children with NEC require surgical intervention. We also established a scientific risk model to predict the timing of surgical intervention under relative surgical indications, laying the theoretical foundation for further improving the clinical identification of high-risk NEC, ensuring timely treatment, preventing further deterioration of the disease, and improving children’s long-term quality of life.

## 2. Materials and Methods

### 2.1. Patient Participants

**Figure 1:**
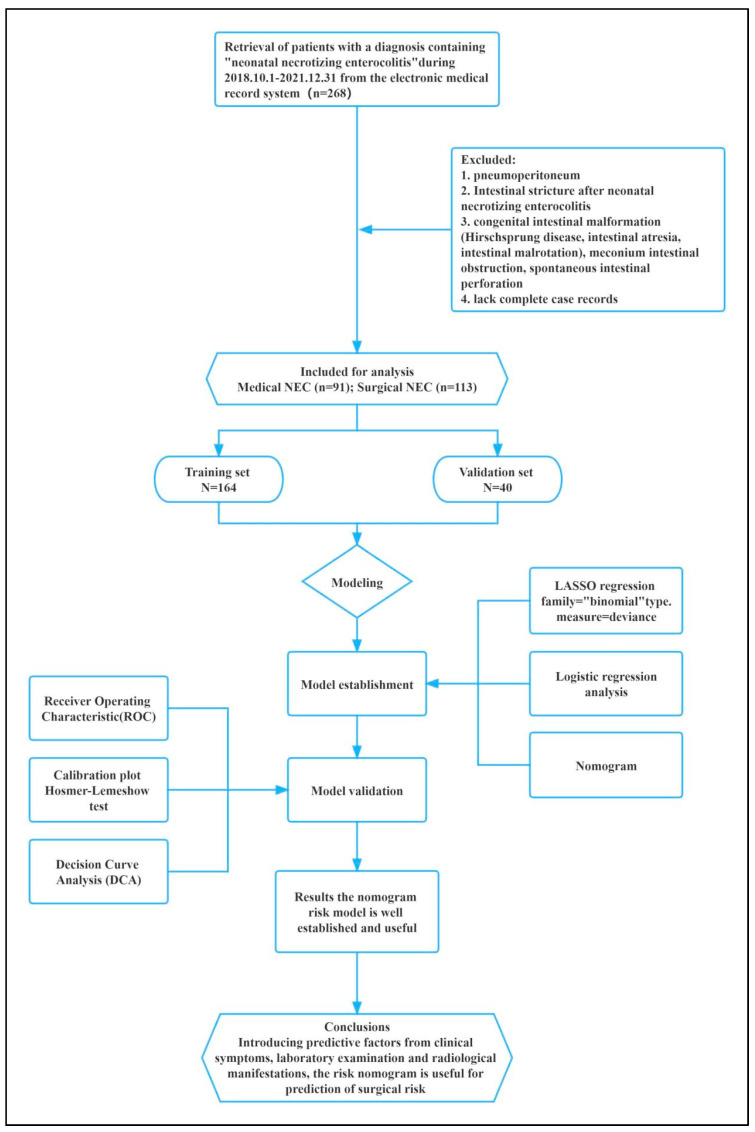
Flow chart of study design. NEC, necrotizing enterocolitis; LASSO, least absolute shrinkage and selection operator; ROC, receiver operating characteristic; DCA, decision curve analysis.

This retrospective cohort analysis included 268 patients diagnosed with NEC at the Children’s Hospital of Zhejiang University School of Medicine between 1 October 2018 and 31 December 2021. We extracted study samples and treatment information from the neonatology, newborn critical care unit, and neonatal surgery department databases. Patients were included in this study if the following criteria were met: (1) Infants were less than 28 days old at onset or were premature infants older than 28 days yet still in the neonatal phase after correction for gestational age (gestational age less than 44 weeks). (2) NEC patients were diagnosed and treated according to modified Bell stage and Walsh grading criteria [9]. (3) During surgery and postoperative pathology, it was determined that the individuals treated surgically had NEC. In contrast, patients with congenital intestinal deformity (congenital megacolon, intestinal atresia, intestinal malrotation), meconium intestinal blockage, spontaneous intestinal perforation, or massive incomplete case records were excluded from this study. The patients with preoperative examination findings of pneumoperitoneum were likewise ruled out, regardless of the presence or absence of NEC. This study was reviewed by the Ethics Committee of the Children’s Hospital affiliated with the Zhejiang University School of Medicine. All parents of children included in this study signed informed consent. A flow diagram of the study design is shown in Figure 1.

### 2.2. Data Collection

Electronic medical records provided demographic and clinical data. Gender, age at onset, number of mother births, gestational age (weeks), birthweight (gram), Apgar scores −1 min, asphyxia, admission time, operation date, and major clinical concurrent symptoms, such as hypothermia, abdominal distension, hematochezia, diarrhea, and vomit were collected. Laboratory parameters include white blood cells (WBC), neutrophils ratio (N%), C-reactive protein (CRP), platelets (PLTs), prothrombin time (PT), activated partial thromboplastin time (APTT), pH, lactic acid (Lac), and imaging results for ileus, pneumatosis intestinalis, intestinal dilatation, intestinal stiffness, ascites/intestinal effusion, and portal pneumatosis.

### 2.3. Statistical Analyses

The R program was used to perform statistical analyses (version 4.2.2; R Foundation for Statistical Computing, Vienna, Austria). The R caret package was used to randomly split the NEC patients into a training set and a validation set in accordance with the theoretical 8:2 ratio. The baseline characteristics were summarized using descriptive statistics. Continuous variables were provided as the median and interquartile range. Categorical information was expressed numerically using percentages. As applicable, Pearson chi-squared tests, the Fisher’s exact test, the Mann–Whitney test, and the McNemar’s test were employed to compare categorical and continuous data between groups. For predictor selection and regularization, LASSO regression analysis was implemented [27]. Lambda.lse produces a model with excellent performance and the fewest independent variables. We incorporated the features chosen in the LASSO regression model, and logistic regression analysis was utilized to develop a prediction model for surgical indication, and a nomogram was constructed based on the model [28]. The characteristics were shown as an odds ratio (OR) and 95% confidence interval (CI). A two-tailed *p* value of less than 0.05 was deemed statistically significant. The receiver characteristic curve (ROC) procedure was utilized to distinguish between real positives and false positives in the surgical risk nomogram [29]. The calibration curves were used to examine the calibration of the surgical risk nomogram, while the decision curve analysis (DCA) was utilized to determine the clinical applicability of the nomogram based on the net benefit under different threshold probabilities.

## 3. Results

### 3.1. Patient Baseline Data

Our study comprised 204 NEC patients; 115 were male and 89 were female. There were 113 individuals who underwent surgery and 91 people who received medicinal treatment. Preterm delivery and low body weight were the most prevalent and significant demographic features of NEC patients. These patients were assigned to the training set or validation set at random. The fundamental characteristics of the two groups of patients are listed in Table 1.

At baseline, the gestational age, birth weight of NEC patients who underwent surgical treatment were significantly lower than those who did not, but the incidence of asphyxia was higher. In terms of clinical symptoms, the number of patients in the surgical therapy group with hypothermia, abdominal distension, and an absence of bowel sounds was relatively high. There were significant variations in WBC, N%, CRP, PLT, PT, APTT, and acidosis between the medicinal therapy and surgical treatment groups, according to the laboratory blood test results. Abdominal X-ray examination results revealed that the surgical therapy group had a greater number of patients with pneumatosis intestinalis, portal pneumatosis, intestinal stiffness and ascites than the medicinal treatment group.

### 3.2. Independent Risk Factors in the Training Set

**Figure 2:**
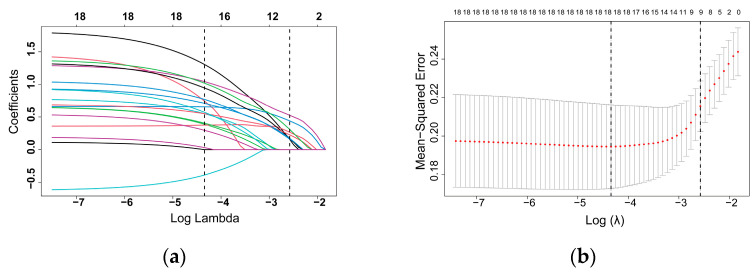
Variable selection with the LASSO model for binary logistic regression. (**a**) The optimal lambda was used to choose eighteen variables with nonzero coefficients. Each line represented a parameter, and the end of the parameter pointed to a vertical coordinate, which was the coefficient. (**b**) Following validation of the optimal parameter (lambda) in the LASSO model, we plotted the partial likelihood deviance (binomial deviance) curve against log (lambda) and created vertical dashed lines based on 1 standard error threshold.

This study comprised 18 variables from clinical symptoms, laboratory examinations, and radiographic manifestations. The log (lambda) sequence was utilized to create a coefficient profile graphic. After confirming the optimal parameter (lambda) in the LASSO model, we plotted the partial likelihood deviance (binomial deviance) curve versus log (lambda) and drew vertical lines with dots based on one standard error (1se) criteria. Using the lambda 1se, nine variables with nonzero coefficients were found (Figure 2).

### 3.3. Predictive Model Construction

**Figure 3:**
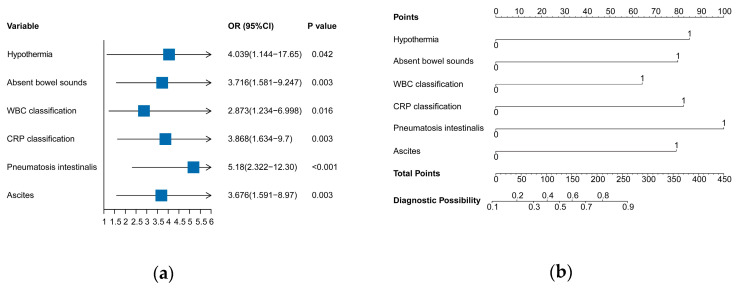
The predictive model was developed using multivariate logistic regression. (**a**) Multivariate logistic regression analysis of surgical intervention risk predictors. (**b**) Nomogram for surgical risk prediction in NEC patients. The odds ratios, confidence intervals, and *p* values are displayed. *p* < 0.05 indicated a statistically significant difference. OR, odds ratio; CI, confidence interval.

The nine predictive variables selected from LASSO regression analysis were used for univariate and multivariate logistic regression (Table 2). Six predictive factors were selected and showed significant statistical difference. They were hypothermia, absent bowel sounds, WBC > 20 × 10^9^/L or < 5 × 10^9^/L, CRP > 50 mg/L, pneumatosis intestinalis, and ascites. The predictive model was established using multivariate logistic regression, and these variables were incorporated into the predictive model to generate a surgical intervention risk nomogram in NEC (Figure 3).

### 3.4. Predictive Model Validation

**Figure 4:**
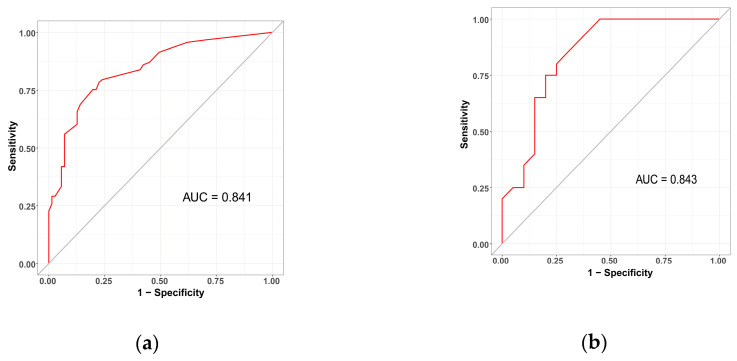
Validation of the surgical intervention risk nomogram using the receiver operating characteristic curve (ROC). The *y*-axis indicates the true positive rate of the risk prediction, while the *x*-axis indicates the false positive rate. The solid red line depicts the performance of the nomogram in the training set (**a**) and validation set (**b**).

The sensitivity and specificity of the predictive model were measured using the ROC curve. The prediction model has an AUC for the nomogram pooled over the training set (AUC = 0.841, cutoff value = 0.578, precision = 0.775, sensitivity = 0.785) and validation set (AUC = 0.843, cutoff value = 0.550, precision = 0.800, sensitivity = 0.750), respectively, indicating fair to good performance (Figure 4).

**Figure 5:**
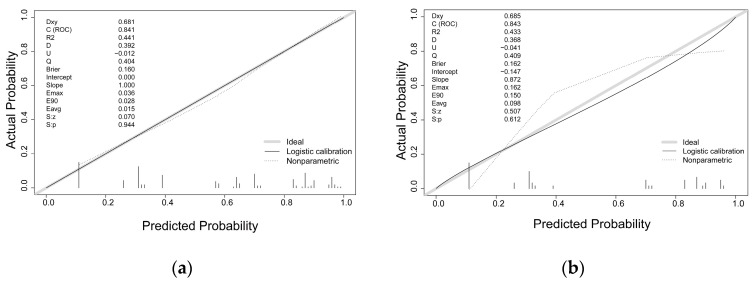
Surgical risk prediction nomogram calibration curves. True surgical cases are plotted along the *y*-axis, while expected surgical intervention risks are shown along the *x*-axis. A better prediction is indicated by a tighter fit to the diagonal dotted line, which represents a perfect prediction by an ideal model, and the solid line, which shows the performance of the training set (**a**) and validation set (**b**).

**Figure 6:**
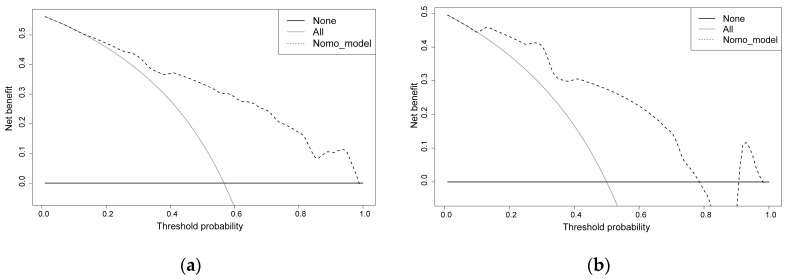
Decision curve analysis for the surgical risk nomogram. The *y*-axis measures the net benefit. The thick solid line represents the assumption that all patients have no surgical intervention, the thin solid line represents the assumption that all patients have surgical intervention, and the dotted line represents the risk nomogram. (**a**) From the training set, (**b**) from the validation set.

The predictive model was calibrated with the use of a calibration curve and Hosmer–Lemeshow test. The calibration curves demonstrated a very good degree of fit for both the predictive model and the validation set. Hosmer–Lemeshow analysis demonstrated excellent agreement between the calculated and the observed probabilities (Figure 5). The nomogram DCA similarly suggested the model might be useful in clinical settings (Figure 6).

## 4. Discussion

Overdiagnosis or delayed diagnosis is an unavoidable issue in children with NEC because of the intricacy of the pathological process, the continuity and progression of the disease, and the lack of specific diagnostic indicators. There has always been a need for careful clinical attention to foresee the unique illness development process and devise therapeutic options that correspond to the optimal time for surgical investigation. In this study, we created a risk prediction model for surgery in NEC children by comparing the pertinent data of children with NEC who received medical treatment with those who had surgical treatment by retrospective analysis. Independent risk factors for surgical intervention risk in children with NEC were hypothermia, absent bowel sounds, WBC > 20 × 10^9^/L or < 5 × 10^9^/L, CRP > 50 mg/L, pneumatosis intestinalis, and ascites. This model can identify the risk of surgery in children with NEC at an early stage, aiding primary doctors to make clinical decisions and providing personalized treatment.

Children with NEC often develop fever in the early stages of infection because bacteria and their toxins can either cause direct damage to the mucosa or indirectly enhance the release of inflammatory mediators. Newborns, especially premature infants, have a relatively large body surface area and less subcutaneous fat, all of which can impair the nerve reflex control and the thermogenesis of brown fat in the event of a serious illness, causing the newborns to display no increase or drop in body temperature. Hypothermia can also cause intestinal vasoconstriction and decreased blood flow. If ischemia persists or ischemia–reperfusion occurs, it can exacerbate intestinal mucosal injury and even cause intestinal necrosis. In this investigation, we confirmed that hypothermia is an independent risk factor for surgical intervention in NEC patients, with the risk of necessitating surgical exploration being four times higher in children with hypothermia than in children without hypothermia. Frequently, children with hypothermia NEC develop severe infections. Necrotic tissue should be removed by surgical exploration if it is evidently associated with the intestine to prevent the contents of the intestinal cavity from entering the abdominal cavity, reduce the inflammation caused by necrotic tissue and intestinal contents, and control the symptoms of infection and poisoning.

An indirect indicator of intestinal activity, bowel sound is produced by the collision of ingested material with the intestinal wall or between individual canals during peristalsis [30]. Bowel sound may diminish or stop altogether in conditions such as intestinal blood circulation disturbance, intestinal necrosis, intestinal paralysis, and severe peritonitis [31]. Abdominal auscultation in children with NEC typically reveals either very busy or feeble bowel sounds. For patients with NEC, the loss of bowel sounds was an independent risk factor for requiring surgical intervention, and it was observed in 41.6% of the NEC group requiring surgery. There is considerable overlap between the symptoms and signs of NEC and those of sepsis and spontaneous intestinal perforation. Consequently, that children with NEC had no bowel sounds on dynamic monitoring suggested intestinal motor function was impaired. Timely evaluation by clinicians, in conjunction with laboratory and imaging studies, is essential, as is the possibility of exploratory surgery [19].

Pathophysiologically, NEC develops when initial-response intestinal inflammation spreads throughout the body. Biochemical changes in the blood are an early sign of NEC, and this inflammatory response worsens over time. Systemic inflammation is linked to more severe cases of NEC. As an acute-phase-response protein, CRP levels begin to rise 10–12 h after infection exposure, continuing to climb for 24–48 h after infection exposure [32]. Whenever there is inflammation due to an infection or tissue damage, inflammatory cytokines drive the liver’s epithelial cells to release a lot of CRP. This results in a sharp rise in CRP concentration. Children with intestinal perforation were more likely to have aberrant WBCs and higher CRP levels when comparing cases with and without perforation [33]. We observed that CRP levels were considerably higher in the NEC surgical treatment group compared with the medical therapy group, and that 44.2% of the NEC surgical group’s children had abnormal WBC values. Consistent with prior research, high levels of CRP and WBC abnormalities are risk factors for necessitating surgical treatment in children with NEC [34,35]. It is normal for the inflammatory index to rise in response to infection and inflammation, but persistently high values are generally indicative of a dangerous illness; in the case of NEC children, this may even include intestinal necrosis and perforation [36,37,38]. As a result, when NEC has occurred, many doctors will constantly examine their patients’ WBC and CRP levels, as well as other inflammatory indicators, to determine how severe the disease is [34,39].

In a clinical setting, an abdominal X-ray examination is of considerable utility for both diagnosing NEC and evaluating disease severity. The DAAS evaluation system semi-quantitatively analyzes abdominal X-ray images [24]. Based on the results of previous research, it appears to be a useful imaging reference for determining whether to operate on children with NEC. Clinical research, however, revealed that many children with NEC exhibited considerable alterations in abdomen plain films prior to intestinal perforation, and that the appraisal of the same NEC children’s abdominal plain films by different clinicians and medical institutions varied to some degree. There is room for error in the diagnosis and surgical management of children with NEC if practitioners rely solely on the DAAS evaluation method. Incorporating traditional imaging markers, we discovered that abdominal X-ray findings in the surgery group’s children were indicative of pneumatosis intestinalis in 50.4% and ascites in 46.9%. Intestinal-wall gas is prevalent and often occurs at the fixed intestinal loop in the advanced stage of NEC, and this is a key indicator of intestinal necrosis known as pneumatosis intestinalis [40]. Intestinal necrosis included the mucosal, submucosal, and muscular layers but spared the serosa. Gas from the intestinal cavity diffused across the subserosa after passing through the necrotic intestinal wall. When the peritoneum is involved in the intestinal wall’s inflammatory necrosis of the intestinal wall, the peritoneal exudates progressively increase, resulting in an increase in abdominal density, bilateral flank abdomens that bulge outward, intestinal loops that float, a widened gap between the gas-filled intestine and the abdominal wall, and a widened gap between the intestines. Therefore, to avoid delaying the best operations, when abdominal X-ray results indicate pneumatosis intestinalis and ascites, clinical symptoms and other test results should be combined in clinical practice rather than merely focusing on whether pneumoperitoneum is present [41].

Single abdominal exam evaluation, imaging exam evaluation, biomarker or serological monitoring data, and the combined application of numerous markers have all been used in previous clinical studies to forecast operation schedules [24,34,42,43,44]. Clinical symptoms, laboratory tests, and imaging studies are the focus of this research because they are the most considerable factors for developing prediction systems for clinical practice. Using LASSO regression analysis, we separated the study’s data into training and validation sets and then utilized the results to screen the inclusion factors and create a nomogram model. The clinical prediction model that was developed has been thoroughly and successfully validated. The prediction model can dynamically monitor and analyze changes in the disease, and it is easily operated in clinical operations, making it desirable for such purposes. However, our clinical prediction model is not perfect because it has not been checked by anyone outside of our own institution. Moreover, the model can be further refined to better meet the demands of clinical pediatricians and surgeons by including specific biomarkers for measuring intestinal necrosis in future clinical studies.

## 5. Conclusions

In conclusion, based on our laboratory examination and analysis of the clinical data and radiological manifestations of NEC patients in our center, we found that hypothermia, absent bowel sounds, WBC > 20 × 10^9^/L or < 5 × 10^9^/L, CRP > 50 mg/L, pneumatosis intestinalis, and ascites were independent risk factors for surgical intervention. The simple and reproducible nomogram clinical prediction model constructed on this premise can assist physicians in evaluating the progress of children with NEC in a timely and effective manner, allowing for the early identification and diagnosis of children at risk for surgery. Therefore, nomogram has clinical transformational value and can play a role in developing treatment plans for the medicinal or surgical treatment of children with NEC.

## Figures and Tables

**Table 1:** Characteristics of the 204 patients with NEC enrolled in the study according to medical NEC and surgical NEC.

	ALL Patients (N = 204)	Medical NEC (N = 91)	Surgical NEC (N = 113)	*p* Value
**Basic information**				
Gender, n (%)				0.140
Male	115 (56.4%)	57 (62.6%)	58 (51.3%)	
Female	89 (43.6%)	34 (37.4%)	55 (48.7%)	
Delivery method, n (%)				0.258
Vaginal	71 (34.8%)	36 (39.6%)	35 (31.0%)	
Cesarean section	133 (65.2%)	55 (60.4%)	78 (69.0%)	
Gestational age (weeks)	32.0 [29.0;35.0]	33.0 [30.0;35.5]	31.0 [28.0;34.0]	0.002
Gestational age (weeks)				0.024
≥37	31 (15.2%)	15 (16.5%)	16 (14.2%)	
32–36	65 (31.9%)	24 (26.4%)	41 (36.3%)	
28–31	81 (39.7%)	45 (49.5%)	36 (31.9%)	
<28	27 (13.2%)	7 (7.69%)	20 (17.7%)	
Birth weight (g)	1735 [1280;2300]	2000 [1550;2412]	1500 [1200;2160]	0.001
Birth weight (g)				0.002
≥2500	38 (18.6%)	19 (20.9%)	19 (16.8%)	
1500–2499	50 (24.5%)	13 (14.3%)	37 (32.7%)	
1000–1499	90 (44.1%)	51 (56.0%)	39 (34.5%)	
<1000 g	26 (12.7%)	8 (8.79%)	18 (15.9%)	
Admission age (d)	13.0 [5.00;23.2]	12.0 [4.00;22.0]	13.0 [8.00;25.0]	0.116
Onset age (d)	11.0 [7.00;19.2]	11.0 [6.50;17.5]	12.0 [7.00;20.0]	0.314
Apgar 1 min, n (%)	9.00 [8.00;10.0]	9.00 [8.50;10.0]	9.00 [8.00;10.0]	0.026
Apgar 1 min classification				0.223
8–10	164 (80.4%)	77 (84.6%)	87 (77.0%)	
4–7	37 (18.1%)	14 (15.4%)	23 (20.4%)	
1–3	3 (1.47%)	0 (0.00%)	3 (2.65%)	
Asphyxia, n (%)				<0.001
No	102 (50.0%)	61 (67.0%)	41 (36.3%)	
Yes	102 (50.0%)	30 (33.0%)	72 (63.7%)	
**Clinical manifestations**				
Hypothermia, n (%)				0.010
No	181 (88.7%)	87 (95.6%)	94 (83.2%)	
Yes	23 (11.3%)	4 (4.40%)	19 (16.8%)	
Abdominal distention, n (%)				0.002
No	46 (22.5%)	30 (33.0%)	16 (14.2%)	
Yes	158 (77.5%)	61 (67.0%)	97 (85.8%)	
Hematochezia, n (%)				0.058
No	45 (22.1%)	14 (15.4%)	31 (27.4%)	
Yes	159 (77.9%)	77 (84.6%)	82 (72.6%)	
Diarrhea, n (%)				1.000
No	199 (97.5%)	89 (97.8%)	110 (97.3%)	
Yes	5 (2.45%)	2 (2.20%)	3 (2.65%)	
Emesis, n (%)				0.056
No	169 (82.8%)	81 (89.0%)	88 (77.9%)	
Yes	35 (17.2%)	10 (11.0%)	25 (22.1%)	
Absent bowel sounds, n (%)				<0.001
No	141 (69.1%)	75 (82.4%)	66 (58.4%)	
Yes	63 (30.9%)	16 (17.6%)	47 (41.6%)	
**Laboratory examination**				
WBC (10^9^/L)	7.84 [4.73;11.2]	8.85 [6.81;11.7]	5.91 [3.24;10.8]	<0.001
WBC classification				<0.001
5–20 × 10^9^/L	137 (67.2%)	74 (81.3%)	63 (55.8%)	
>20 × 10^9^/L or <5 × 10^9^/L	67 (32.8%)	17 (18.7%)	50 (44.2%)	
N, n (%)	53.1 [40.3;69.6]	44.1 [34.0;61.5]	59.3 [46.9;71.8]	<0.001
N classification				0.041
≤75%	176 (86.3%)	84 (92.3%)	92 (81.4%)	
>75%	28 (13.7%)	7 (7.69%)	21 (18.6%)	
CRP (mg/L)	23.5 [1.39;62.8]	1.69 [0.50;22.5]	50.0 [21.2;88.5]	<0.001
CRP classification				<0.001
<50 mg/L	134 (65.7%)	78 (85.7%)	56 (49.6%)	
≥50 mg/L	70 (34.3%)	13 (14.3%)	57 (50.4%)	
PLT (10^9^/L)	202 [118;302]	245 [184;367]	177 [103;246]	<0.001
PLT classification				0.006
≥100 × 10^9^/L	171 (83.8%)	84 (92.3%)	87 (77.0%)	
<100 × 10^9^/L	33 (16.2%)	7 (7.69%)	26 (23.0%)	
PT * (s)	14.1 [12.9;17.0]	13.6 [12.8;14.9]	15.1 [13.2;18.3]	<0.001
PT * classification				<0.001
<17 s	140 (74.1%)	68 (88.3%)	72 (64.3%)	
≥17 s	49 (25.9%)	9 (11.7%)	40 (35.7%)	
APTT * (s)	53.0 [46.1;74.4]	49.5 [40.3;59.7]	58.0 [49.2;80.6]	<0.001
APTT * classification				0.002
<70 s	138 (73.0%)	66 (85.7%)	72 (64.3%)	
≥70 s	51 (27.0%)	11 (14.3%)	40 (35.7%)	
pH	7.37 [7.31;7.42]	7.40 [7.36;7.44]	7.34 [7.25;7.40]	<0.001
Acidosis (pH < 7.35)				<0.001
No	125 (61.3%)	71 (78.0%)	54 (47.8%)	
Yes	79 (38.7%)	20 (22.0%)	59 (52.2%)	
Lac (mmol/L)	2.40 [1.50;3.40]	2.40 [1.75;3.25]	2.30 [1.40;3.50]	0.377
Lac classification				0.079
≤1.6 mmol/L	61 (29.9%)	21 (23.1%)	40 (35.4%)	
>1.6 mmol/L	143 (70.1%)	70 (76.9%)	73 (64.6%)	
**Radiological manifestations**				
Intestinal dilation, n (%)				0.057
No	57 (27.9%)	32 (35.2%)	25 (22.1%)	
Yes	147 (72.1%)	59 (64.8%)	88 (77.9%)	
Ascites, n (%)				0.001
No	129 (63.2%)	69 (75.8%)	60 (53.1%)	
Yes	75 (36.8%)	22 (24.2%)	53 (46.9%)	
Intestinal stiffness, n (%)				<0.001
No	104 (51.0%)	60 (65.9%)	44 (38.9%)	
Yes	100 (49.0%)	31 (34.1%)	69 (61.1%)	
Pneumatosis intestinalis, n (%)				<0.001
No	126 (61.8%)	70 (76.9%)	56 (49.6%)	
Yes	78 (38.2%)	21 (23.1%)	57 (50.4%)	
Ileus, n (%)				0.741
No	158 (77.5%)	69 (75.8%)	89 (78.8%)	
Yes	46 (22.5%)	22 (24.2%)	24 (21.2%)	
Portal pneumatosis, n (%)				0.021
No	177 (86.8%)	85 (93.4%)	92 (81.4%)	
Yes	27 (13.2%)	6 (6.59%)	21 (18.6%)	

Notes: *p* < 0.05 meant that the difference was statistically significant. Abbreviations: WBC, white blood cells; CRP, C-reactive protein; PT, prothrombin time; APTT, activated partial thromboplastin time; Lac, lactic acid; * Represent have missing value.

**Table 2:** Univariate and multivariate logistic regression analysis were performed to screen the predictors for the surgical risk from LASSO regression analysis.

Characteristics	Uni-B	Uni-SE	Uni-OR	Uni-CI	Uni-P	Multi-B	Multi-SE	Multi-OR	Multi-CI	Multi-P
Hypothermia	1.247	0.58345	3.481	1.109–10.922	0.033	1.71	0.76935	5.527	1.224–24.968	0.026
Absent bowel sounds	1.214	0.37153	3.367	1.626–6.975	0.001	1	0.49527	2.717	1.029–7.173	0.044
WBC classification	1.123	0.36447	3.073	1.504–6.277	0.002	0.993	0.49895	2.698	1.015–7.175	0.047
CRP classification	1.632	0.38811	5.114	2.39–10.942	<0.001	1.089	0.50494	2.972	1.105–7.996	0.031
PT classification	1.544	0.45618	4.684	1.915–11.452	0.001	0.867	0.57066	2.38	0.778–7.285	0.129
Acidosis	1.603	0.37054	4.969	2.403–10.272	<0.001	0.903	0.47685	2.468	0.969–6.284	0.058
Ascites	1.048	0.3471	2.852	1.445–5.632	0.003	1.301	0.50546	3.671	1.363–9.887	0.01
Intestinal stiffness	1.087	0.32837	2.964	1.557–5.641	0.001	0.8	0.44533	2.225	0.93–5.326	0.073
Pneumatosis intestinalis	1.299	0.35176	3.667	1.84–7.306	<0.001	1.347	0.46464	3.845	1.547–9.56	0.004

Notes: Odds ratios, confidence intervals, and *p*-values were shown. *p* < 0.05 meant that the difference was statistically significant. Abbreviations: WBC, white blood cells; CRP, C-reactive protein; PT, prothrombin time; OR, odds ratio; CI, confidence interval.

## Data Availability

Not applicable.
